# A Study of the Effects of Doxorubicin-Containing Liposomes on Osteogenesis of 3D Stem Cell Spheroids Derived from Gingiva

**DOI:** 10.3390/ma12172693

**Published:** 2019-08-23

**Authors:** Hyunjin Lee, Jihwan Son, Sae Kyung Min, Chae-Bin Na, Gawon Yi, Heebeom Koo, Jun-Beom Park

**Affiliations:** 1 Department of Periodontics, College of Medicine, The Catholic University of Korea, Seoul 06591, Korea; 2 Department of Medical Life Sciences and Department of Biomedicine & Health Sciences, College of Medicine, The Catholic University of Korea, Seoul 06591, Korea

**Keywords:** cell differentiation, cellular spheroids, doxorubicin, gingiva, stem cells

## Abstract

The objective of the present investigation is to determine the effects of neutral, anionic, and cationic liposomes loaded with doxorubicin with thin-lipid-film-hydration method on the cellular viability and osteogenesis of stem cell spheroids. Spheroid formation and morphology of the three-dimensional spheroid were noted with an inverted microscope. Quantitative cellular viability was assessed using a commercially available kit. Osteogenic potential was evaluated by applying alkaline phosphatase activity and anthraquinone dye of Alizarin Red S. Western blot analysis was performed using collagen I expression. Spheroids were formed in each silicon elastomer-based concave microwell on Day 1. Noticeable changes of the spheroid were seen with a higher concentration of doxorubicin, especially in the cationic liposome group at Days 5 and 7. We found that the application of doxorubicin for 5 days significantly reduced the cellular viability. A higher concentration of doxorubicin produced a significant decrease in alkaline phosphatase activity. Alizarin Red S staining showed that extracellular calcium deposits were evenly noted in each group. An increase of calcium deposits was noted on Day 14 when compared to Day 7. The morphology of the groups with higher concentrations of doxorubicin showed to be more dispersed. We noticed that doxorubicin-loaded cationic liposomes resulted in the highest uptake of the examined cell spheroids and that doxorubicin-loaded liposomes affected the osteogenic differentiation. The implication of this study is that the type of liposome should be selected based on the purpose of the application.

## 1. Introduction

Doxorubicin was originally isolated from *Streptomyces peucetius* [1]. Doxorubicin is an anthracycline chemotherapy agent and is shown to have in vitro and in vivo anti-tumor activities [2]. Localized delivery of doxorubicin is shown to be effective for the treatment of prostate cancer cells [3]. Common side effects for chemotherapy include bone marrow suppression, hair loss, rash, and inflammation of the mouth [4], Doxorubicin is shown to have a dose-related cardiotoxicity and this can lead to heart failure in a subset of patients [5]. Mesenchymal stem cells have been used for the treatment of cardiotoxicity, and stem cells have significantly ameliorated the cardiotoxic manifestations through functional, structural, and biochemical cardiac improvement [6].

A tissue-engineered three-dimensional microenvironment enhances the direct reprogramming when compared with the traditional two-dimensional culture [7]. It is also shown that three-dimensional spatial boundary environments control osteogenesis of mesenchymal stem cells [8]. Doxorubicin is reported to have adverse effects on bone turnover, especially on osteoblastic activity [9]. A previous report showed that participants undergoing chemotherapy with doxorubicin may experience inhibitory effects [10]. Cytotoxicity of doxorubicin is an advantage as an anticancer drug, but it may it may have an influence on the differentiation potential of stem cells during chemotherapy. In particular, the effects of doxorubicin on three-dimensional stem cell cultures are not well revealed yet. Moreover, liposomes are reported to be an optimal drug delivery system for doxorubicin [11]. Gingiva-derived stem cells can be obtained from daily practice in dental clinics and they have good osteogenic potentials [12,13,14]. Gingiva-derived stem cells produce growth factors and express stem cell surface makers of CD44, CD73, CD90 and CD105 [15,16]. The objective of present study is to evaluate the effects of doxorubicin-containing neutral, anionic, and cationic liposomes on the viability and osteogenesis of cell spheroids made from human gingiva-derived stem cells.

## 2. Materials and Methods

### 2.1. Preparation of Doxorubicin-Containing Liposomes

We prepared liposomes using the thin-lipid-film-hydration method following previous publications [17,18]. In brief, the lipids were dissolved in dichloromethane (Daejung, Siheung-si, Gyeonggi-do, Korea), and the solvent was removed. Then, the film of lipids was dispersed in the distilled water containing doxorubicin hydrochloride (LC laboratories, Woburn, MA, USA) by sonication. Then, removal of unloaded doxorubicin was done through dialysis for one hour. We analyzed the amount of doxorubicin in the liposomes by measuring the fluorescence of doxorubicin (490/570 nm) after liposomes were completely disassembled by Triton X-100 (Samchun, Pyeongtaek-si, Gyeonggi-do, Korea).

### 2.2. Release Profile of Doxorubicin

The release of doxorubicin from the liposomes was evaluated in phosphate-buffered saline at room temperature. We loaded doxorubicin-containing liposomes in a dialysis bag, and measured the amount of remaining doxorubicin based on the fluorescence.

### 2.3. Formation of Cell Spheroids with Human Gingiva-Derived Stem Cells

The Institutional Review Board reviewed of Seoul St. Mary’s Hospital, College of Medicine, Catholic University of Korea, Seoul, Republic of Korea gave approval for the study (KC17SESI0290). All the procedures were performed following the relevant guidelines and regulations. Gingival tissues were collected and epithelium was removed. The tissues were cut into small pieces of 1–2 mm^2^ and digestion was done with enzymes (dispase and collagenase IV). Stem cell spheroids used gingiva-derived stem cells in the amount of 9 × 10^5^ cells using the silicon elastomer-based concave microwells (H389600, StemFIT 3D; MicroFIT, Seongnam, Korea) having 600 μm diameters. This study had twelve groups of (1) an unloaded control group (Doxo0); (2) doxorubicin at 1 μg/mL (Doxo1); (3) doxorubicin at 10 μg/mL (Doxo10); (4) anionic liposomes without doxorubicin (A0); (5) anionic liposomes containing 1 μg/mL doxorubicin (A1); (6) anionic liposomes containing 10 μg/mL doxorubicin (A10); (7) cationic liposomes without doxorubicin (C0); (8) cationic liposomes containing 1 μg/mL doxorubicin (C1); (9) cationic liposomes containing 10 μg/mL doxorubicin (C10); (10) neutral liposomes without doxorubicin (N0); (11) neutral liposomes containing 1 μg/mL doxorubicin (N1); and (12) neutral liposomes containing 10 μg/mL doxorubicin (N10).

### 2.4. Evaluation of Spheroid Morphology and Determination of Quantitative Cell Viability

Stem cell spheroids were cultured in the osteogenic media (α-MEM supplemented with 15% FBS (Gibco, Grand Island, NY, USA), 200 mM L-glutamine (Sigma-Aldrich Co., St. Louis, MO, USA), 10 mM of ascorbic acid 2-phosphate (Sigma-Aldrich Co.), 38 ug/mL of dexamethasone, 2 mg/mL of glycerophosphate disodium salt hydrate, and 100 U/mL penicillin, and 100 μg/mL streptomycin (Sigma-Aldrich Co.) and the media were replaced with fresh media every 2 to 3 days. We observed spheroid formation and morphology of the cell spheroid on Days 1, 3, 5, and 7 using an inverted microscope (Leica DM IRM, Leica Microsystems, Wetzlar, Germany). We analyzed the quantitative cellar viability using a commercially available kit (CCK-8; Dojindo, Tokyo, Japan) on Days 1, 3, 5, and 7.

### 2.5. Uptake of Doxorubicin by Cell Spheroids with Human Gingiva-Derived Stem Cells

Stem cell spheroids were treated with anionic liposomes loaded with doxorubicin at 10 μg/mL, cationic liposomes loaded with doxorubicin at 10 μg/mL, and neutral liposomes loaded with doxorubicin at 10 μg/mL for 1 h. The unloaded group served as a control. The nuclear counterstain was performed with Hoechest 33342 (Molecular Probes, Inc., Eugene, OR, USA) for 1 h and 30 min.

### 2.6. Osteogenic Differentiation Using Alkaline Phosphatase Activity Assays and Alizarin Red S Staining

We performed the alkaline phosphatase activity assays using a kit (K412-500, BioVision, Inc., Milpitas, CA, USA) on Days 1, 5, and 7 following the manufacturer’s protocol. In short, the cell spheroids were suspended in an assay buffer, sonicated, and centrifuged. Then the supernatant was mixed with a p-nitrophenylphosphate substrate and incubation was performed. The optical density of the resultant p-nitrophenol was measured spectrophotometrically at 405 nm. We performed Alizarin Red S staining on Days 7 and 14.

### 2.7. Western Blot Analysis

We washed the cultures two times and solubilized them using lysis buffer for 30 min on Day 7. We centrifuged the lysates at 15,000 rpm for 10 min at 4 °C. These samples were separated, transferred to the membranes (Immun-Blot^®^, Bio-Rad, Hercules, CA, USA), and blotted with the corresponding antibodies. Primary antibodies against collagen I (ab6308) and glyceraldehyde 3-phosphate (GAPDH; ab9485) and secondary antibodies were purchased from Abcam (Cambridge, UK) and Thermo Fisher Scientific, Inc. (Waltham, MA, USA). Image processing and analyzing programs (ImageJ, National Institutes of Health, Bethesda, MD, USA) were used to analyze the protein expression.

### 2.8. Statistical Analysis

The results were expressed as means ± standard deviations of the experiments. A test of normality of the samples was performed. The non-parametric Mann–Whitney and Kruskal–Wallis test were used to test for statistical significances between the control and the test groups with the statistical program (SPSS 12 for Windows, SPSS Inc., Chicago, IL, USA). We set statistical significance at *p* < 0.05.

## 3. Results

### 3.1. Release Profile of Doxorubicin

**Figure 1:**
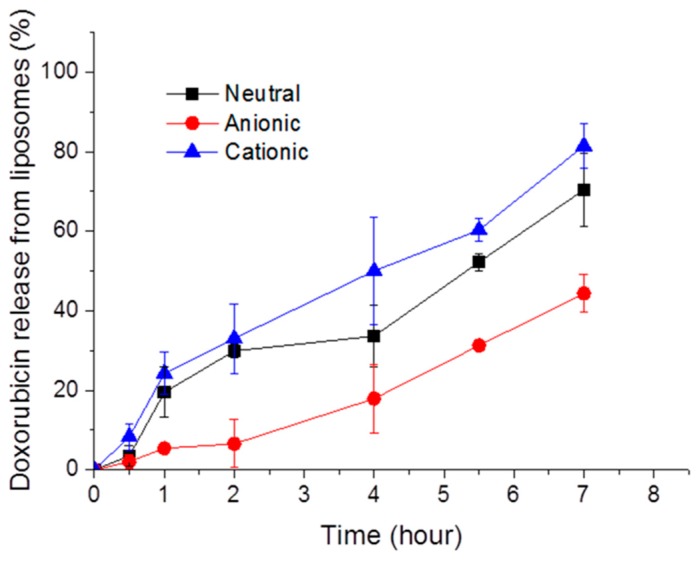
Time-dependent release of doxorubicin from neutral, anionic, and cationic liposomes.

We prepared anionic, cationic, and neutral liposomes containing doxorubicin using the traditional film hydration method. Also, we observed that doxorubicin was gradually released from liposomes as time went on (Figure 1). Anionic liposomes showed slower release than the neutral or cationic ones, possibly due to the interaction with amine groups in doxorubicin.

### 3.2. Cellular Morphology and Cellular Viability

**Figure 2:**
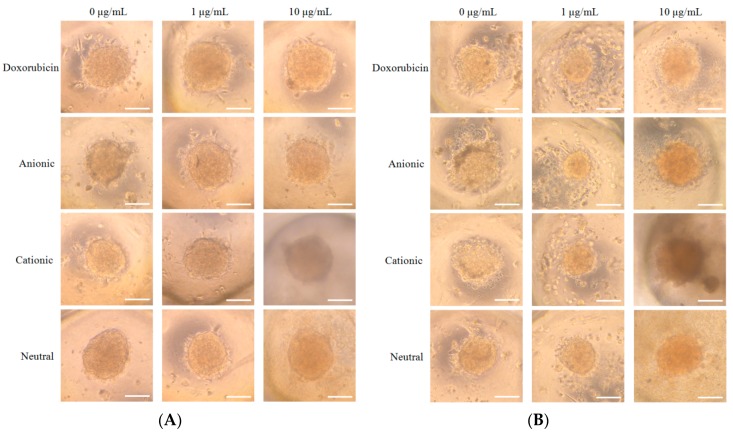

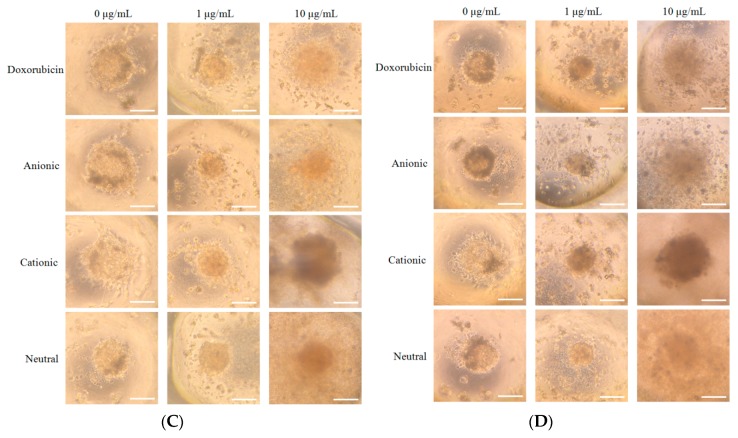
(**A**) Morphology of the cell spheroids cultured in osteogenic media on Day 1 (original magnification ×200). The scale bar indicates 100 μm. (**B**)**.** Morphology of the cell spheroids cultured in osteogenic media on Day 3 (original magnification ×200). The scale bar indicates 100 μm. (**C**) Morphology of the cell spheroids cultured in osteogenic media on Day 5 (original magnification ×200). The scale bar indicates 100 μm. (**D**) Morphology of the cell spheroids cultured in osteogenic media on Day 7 (original magnification ×200). The scale bar indicates 100 μm.

We noticed spheroid formation in the microwells on Day 1 (Figure 2A). The addition of doxorubicin or doxorubicin-loaded liposomes did not produce significant change in morphology on Day 1 (Figure 2A). The morphology results of Days 3, 5, and 7 are shown in Figure 2B–D, respectively. In general, the morphology of the cells in the tested groups was similar to the shapes of the cells in the control group, except for the 10 μg/mL group. Significant changes in the morphology were noted for C10 at Day 3 (Figure 2B). The shapes of the cells in C10 at Day 5 were larger, and cells were adrift (Figure 2C). The qualitative viability using CCK-8 results on Days 1, 3, 5, and 7 is shown in Table 1.

### 3.3. Uptake of Doxorubicin by Cell Spheroids with Human Gingiva-Derived Stem Cells

**Figure 3:**
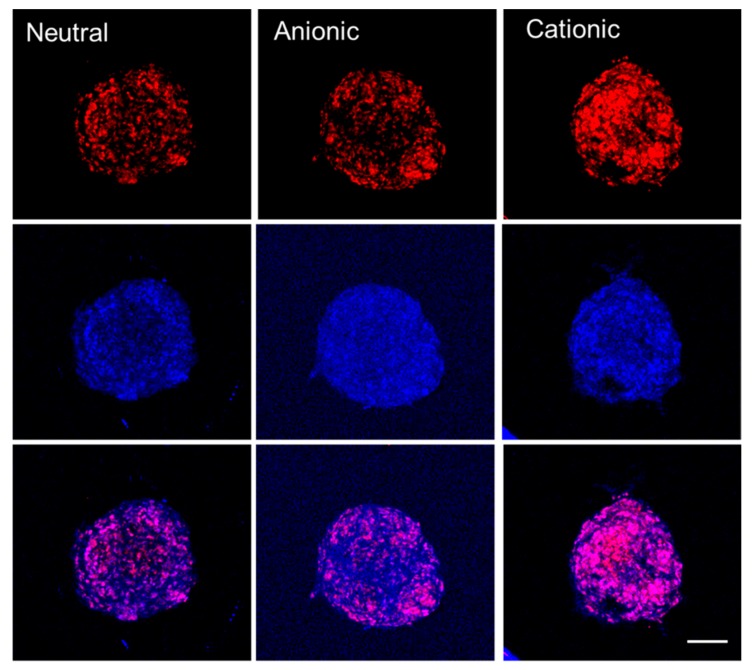
Cellular uptake of neutral, anionic, and cationic liposomes into spheroids. Red = doxorubicin. Blue = DAPI. Scale bar = 100 μm.

When we observed the uptake of three liposomes containing doxorubicin into spheroids, cationic liposomes showed the most intense red fluorescence, meaning the highest uptake (Figure 3).

### 3.4. Evaluation of Osteogenic Differentiation

**Figure 4:**
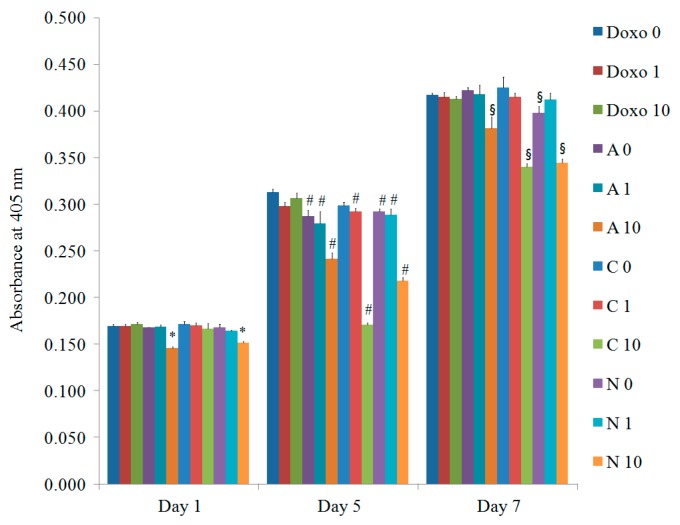
The alkaline phosphatase activity on Days 1, 5, and 7. * Statistically significant differences were noted when compared with the data from the doxorubicin 0 μg/mL (Doxo0) group on Day 1 (*p* = 0.015). # Statistically significant differences were noted when compared with the data from the doxorubicin 0 μg/mL (Doxo0) group on Day 5 (*p* = 0.001). § Statistically significant differences were noted when compared with the data from the doxorubicin 0 μg/mL (Doxo0) group on Day 7 (*p* = 0.003).

The alkaline phosphatase activity treated with doxorubicin on Days 1, 5, and 7 is described in Figure 4 (*n* = 3). The increase of alkaline phosphatase activity was noted with a longer incubation time. The relative values on Day 7 for Doxo0, Doxo1, Doxo10, A0, A1, A10, C0, C1, C10, N0, N1, and N10 were 245.8% ± 1.2%, 244.6% ± 2.7%, 243.4% ± 1.6%, 248.7% ± 1.8%, 246.4% ± 5.7%, 224.6% ± 7.2%, 250.3% ± 6.9%, 244.4% ± 2.8%, 200.2% ± 2.4%, 234.6% ± 4.1%, 242.8% ± 4.1%, and 202.9% ± 2.4%, respectively, when the value of Doxo0 at Day 1 was considered as 100% (100.0% ± 1.2%). A statistically significant decrease of alkaline phosphatase activity was observed among the 10 μg/mL doxorubicin-loaded liposome groups (A10, C10, and N10) on Days 5 and 7 when compared with the doxorubicin unloaded control group (Doxo0) in each group (*p* < 0.05).

**Figure 5:**
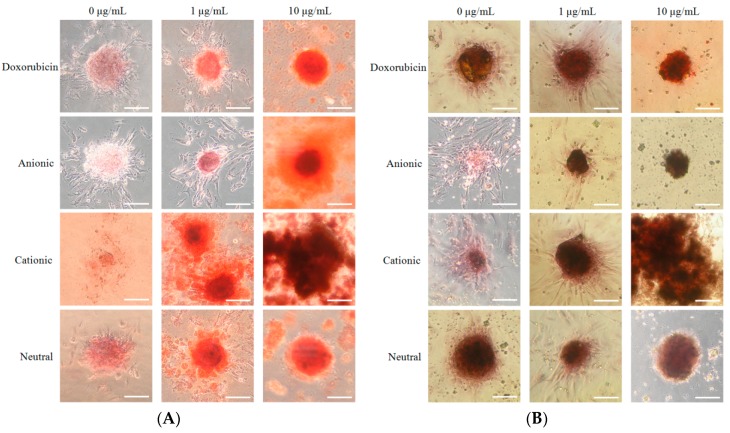
(**A**) The results of the Alizarin Red S staining on Day 7 (original magnification ×200). The scale bar indicates 100 μm. (**B**) The results of the Alizarin Red S staining on Day 14 (original magnification ×200). The scale bar indicates 100 μm.

We analyzed the mineralized extracellular deposits by evaluating calcium on Days 7 and 14 (Figure 5A,B). We noticed even mineralized extracellular deposits in each group. An increase of mineralized deposits was noted on Day 14 when compared to Day 7. However, the morphology of the C10 group showed to be more dispersed.

### 3.5. Western Blot Analysis

**Figure 6:**
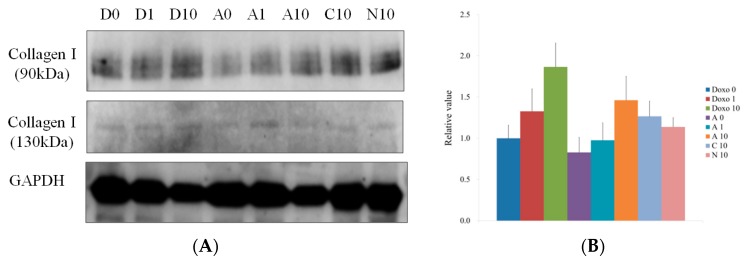

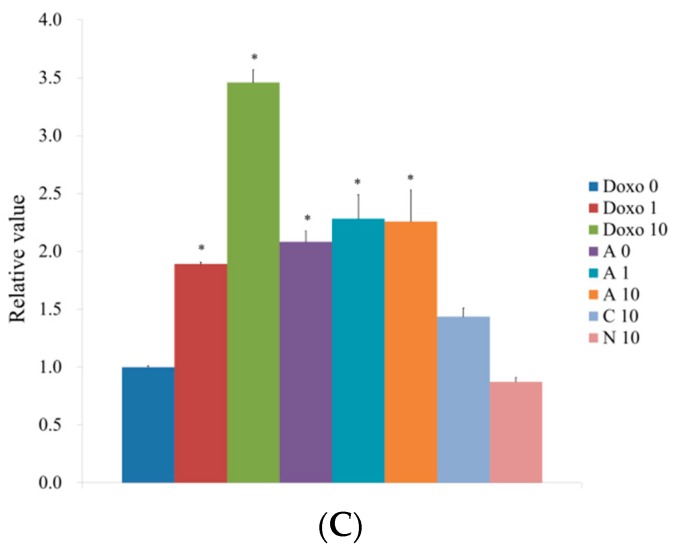
(**A**) Western blot analysis to detect the collagen I protein expressions of spheroids grown in osteogenic media on Day 7. (**B**) Quantitative results of Western blot analysis of collagen I (90 kDa). (**C**) Quantitative results of Western blot analysis of collagen I (130 kDa). * Statistically significant differences were seen when compared with the data from the doxorubicin 0 μg/mL (Doxo0) group (*p* < 0.05).

The Western blot data for collagen I (90 kDa and 130 kDa) on Day 7 are shown in Figure 6A. The protein expression normalizations of collagen I (90 kDa) on Day 7 for Doxo0, Doxo1, Doxo10, A0, A1, A10, C10, and N10 were 100.0%, 132.8%, 186.7%, 82.9%, 97.6%, 146.3%, 126.7%, and 113.8%, respectively (Figure 6B). The protein expression normalizations of collagen I (130 kDa) on Day 7 for Doxo0, Doxo1, Doxo10, A0, A1, A10, C10, and N10 were 100.0%, 189.1%, 345.9%, 208.2%, 228.3%, 225.8%, 143.5%, and 87.3%, respectively (Figure 6C).

## 4. Discussion

This study was aimed to demonstrate the effects of doxorubicin-containing neutral, anionic, and cationic liposomes on the cellular viability and osteogenesis of cell spheroids. Highest uptake by cell spheroids was seen in cationic liposomes with doxorubicin, and a higher concentration of doxorubicin-containing liposomes affected the osteogenic differentiation.

It was shown that doxorubicin induced decreases of osteoblast survival and differentiation in the previous report [19]. Significant changes were observed from 10 μg/mL groups, so that we used this concentration for experiments in this study. This study clearly showed that alkaline phosphatase activity was mostly decreased in the highest concentration of doxorubicin, with 91.4% ± 2.9% for the A10 group, 81.5% ± 1.0% for the C10 group, and 82.6% ± 1.0% for the N10 group on Day 7, with the untreated control group being considered 100%. Higher concentrations of doxorubicin seemed more dispersed, possibly due to a loss of cell-to-cell and cell–matrix interactions [20].

Osteogenesis of mesenchymal stem cells can be accessed using various methods. Alkaline phosphatase is reported to be released from the osteoblast [21], and alkaline phosphatase activity is reported to be an early indicator of osteogenic differentiation [22,23]. This study clearly showed that a higher concentration led to a decrease in alkaline phosphatase activity. Calcium deposition happened after matrix maturation, and Alizarin Red S staining was considered a relatively late marker of osteogenic differentiation [24].

The results suggest that the formulation of liposomes influenced the effects of three-dimensional stem cell spheroids. It should be noted that there could be variations in the response of cell spheroids depending on the source of the cells, stage of the cultured cells, duration of the culture, and types of models [25,26].

## 5. Conclusions

This study demonstrated that doxorubicin-loaded cationic liposomes produced the highest uptake of doxorubicin by the cell spheroids. A higher concentration of doxorubicin-loaded liposomes affected the cellular viability and osteogenic differentiation. The implication of this study is that the type of liposome should be selected based on the purpose of the application.

## Figures and Tables

**Table 1:** The cellular viability of stem cell spheroids on Days 1, 3, 5, and 7.

Day	Groups (*n* = 3)												*p*-Value
	Doxo0	Doxo1	Doxo10	A0	A1	A10	C0	C1	C10	N0	N1	N10	
1	1.976 ± 0.060	1.902 ± 0.032	1.887 ± 0.039	2.084 ± 0.021	1.994 ± 0.037	1.949 ± 0.022	2.181 ± 0.022	2.194 ± 0.022	2.018 ± 0.041	2.060 ± 0.075	2.077 ± 0.024	2.137 ± 0.048	0.001
3	0.335 ± 0.005	0.317 ± 0.004	0.311 ± 0.003	0.322 ± 0.001	0.308 ± 0.001	0.287 ± 0.001	0.344 ± 0.001	0.311 ± 0.001	0.363 ± 0.001	0.318 ± 0.004	0.314 ± 0.002	0.308 ± 0.002	0.000
5	0.372 ± 0.002	0.265 ± 0.005	0.233 ± 0.030	0.273 ± 0.011	0.264 ± 0.006	0.261 ± 0.002	0.267 ± 0.003	0.257 ± 0.008	0.251 ± 0.002	0.262 ± 0.003	0.244 ± 0.004	0.243 ± 0.007	0.007
7	0.236 ± 0.003	0.229 ± 0.003	0.230 ± 0.005	0.237 ± 0.003	0.236 ± 0.001	0.218 ± 0.002	0.238 ± 0.002	0.222 ± 0.003	0.225 ± 0.003	0.230 ± 0.003	0.221 ± 0.002	0.227 ± 0.001	0.001
